# Professor Hugh Gurling MBBS

**DOI:** 10.1192/pb.bp.113.046219

**Published:** 2014-02

**Authors:** David Curtis, Jonathan Pimm

## MRCPsych, FRCPsych, MPhil, MD, Professor of Molecular Psychiatry, UCL, honorary consultant psychiatrist, Camden and Islington NHS Foundation Trust

**Figure:**
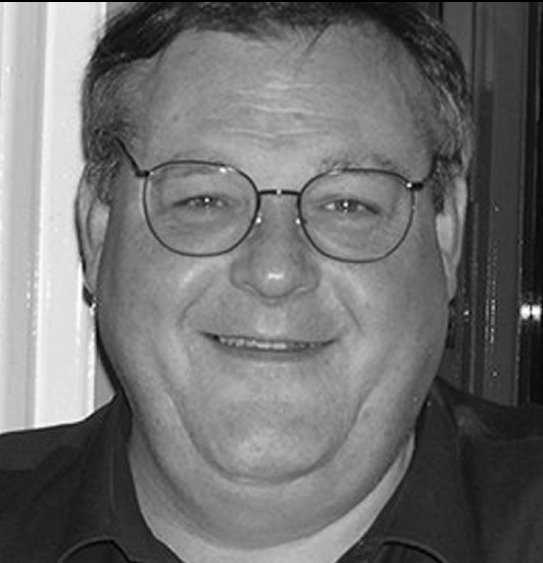


Professor Hugh Gurling, who died aged 63, was a pioneering psychiatrist who was determined to find the genes causing severe mental illness. His resolve to investigate the biological processes underlying disorders such as schizophrenia, manic depression and alcoholism was very much out of kilter with the rest of his academic colleagues when he began his quest in the late 1970s; the focus in psychiatry at that time had been on psychological and sociological factors. However, despite scepticism and at times frank opposition, Hugh single-handedly obtained funding to develop and lead a research unit at University College London, an establishment that was to become a centre recognised worldwide as being at the forefront of psychiatric genetics.

During the course of his career, and due in no small measure to his drive and enthusiasm, the field flourished to the extent that it now engages hundreds of researchers worldwide; it has become the aspect of psychiatric research regarded by many as holding the most promise to yield real biological insights and advances in treatment for severe mental illnesses.

Hugh began his research career studying alcoholism under the supervision of Professor Robin Murray at the Institute of Psychiatry in 1976. As part of this project, he sought to uncover genetic influences of alcoholism and this would often involve him driving around London collecting blood samples, meeting pilots at airports, and sometimes visiting patients' houses late at night.

With the emergence of the ‘new genetics’ he realised the potential to gain radical insights into the understanding of schizophrenia and bipolar disorder. To this end, he formed collaborations with researchers in Iceland, where large family sizes and good genealogical records provided ideal material for his purpose. He began studying them with novel technologies to identify DNA markers for disorders.

A statistics course at the London School of Hygiene and Tropical Medicine in 1977 and a year as a Visiting Scholar and Wellcome Training Fellow at Stanford University's Department of Genetics in 1981 were invaluable for equipping him with the necessary skills to take his career on to the next stage.

He was awarded a Wellcome Senior Fellowship and in 1987 he moved to University College London, where he was to remain until his death. In the Molecular Psychiatry Laboratory, which he set up there, he and his team of dedicated researchers drove the field forward.

In 1988, he published a landmark paper in the journal *Nature* which appeared to demonstrate that chromosome 5 harboured a gene for schizophrenia. Although the exact implications of this paper are disputed to this day, it served as a stimulus which spurred the development of innovative techniques needed to tackle the challenges posed by these mental illnesses.

His laboratory localised and cloned genes coding for proteins acting as receptors for neurotransmitters thought to be intrinsically involved in psychosis. He published a paper showing that highly informative markers called microsatellites could be used in gene mapping studies. And subsequently it became standard practice to use panels of these markers to localise the genes responsible for hundreds of genetic disorders.

As the field continued to progress and new technologies emerged, true to form he seized on them enthusiastically. He was involved in work showing that small deletions or duplications of parts of chromosomes could cause schizophrenia. He published papers showing that changes to single DNA bases appeared to dramatically affect the risk of schizophrenia and bipolar disorder. And most recently, he sequenced all 3 billion DNA bases of 100 individuals with bipolar disorder and identified a number of changes which might be causing the disorder. In order to confirm these findings, he arranged for these variants to be typed in samples of thousands of people with and without bipolar disorder. The results have the potential to identify definitively which variants in which genes could cause the disorder. He died before receiving them.

Hugh adopted the title Professor of Molecular Psychiatry arguing that the term ‘psychiatric genetics’ would soon become out of date once the genes were found and research switched to the molecular level of personalised medicine. He proposed that all the interrelated disciplines underpinning this rapidly developing branch of psychiatry, including genetics, proteomics, psychopharmacology and gene therapy, should be referred to collectively as molecular psychiatry. His friendly broadmindedness was well known and researchers and collaborators from a wide variety of backgrounds were attracted to work with him. Despite being appointed professor, he remained modest, approachable and down to earth, insisting everyone call him by his first name.

He was a large, strong man with powerful hands and an intellect and personality to match his size. He could be relied on to deliver a spell-binding lecture at anxiety-provokingly short notice. He would often run out of time as he tried to cram in details of his journey of exploration of the human genome from its infancy through to the present day: audiences were left both fascinated and confused, struggling to keep up, but never bored.

Hugh was born in Kingston upon Thames. His father Kenneth Gurling was a physician and Inaugural Dean of Medicine at the University of Nottingham. His mother Nonie Sempill was a nurse. He attended Sutton Valence School. He was captain of athletics at school and later excelled at many sports including fives, squash, rugby, windsurfing and hammer throwing. He had many interests and was particularly passionate about listening to music. He played several musical instruments including the sitar and dilruba.

He qualified in medicine at King's College London, and began his training in psychiatry first at Guy's Hospital and then at the Maudsley. He worked as a research scientist and Honorary Senior Registrar at the Institute of Psychiatry for a further 2 years before spending a year at Stanford and then finally moving to University College London. He maintained his clinical skills throughout his career, based for the last 23 years on a psychiatric intensive care unit in north London. The NHS has lost another of its fervent supporters and his extraordinary skill, kindness and dedication will be missed by his patients and the ward team.

Hugh was a keen follower of current affairs and held strong political views about integrity and social justice. He had a special sympathy for the difficulties faced by mentally ill people and the disadvantaged in society. His enthusiasm for challenging the status quo extended to his approach to psychiatry and medical research. His didactic and pedagogical style of supervising his many PhD and MD students was inspirational and at times anarchic. He was often heard informing researchers that they ought not to bother regurgitating information from textbooks but instead to focus on their own experiments. He believed they were the future and what they were doing would re-write the history of psychiatry.

He leaves his wife of 26 years, Meryl Dahlitz, also an academic psychiatrist with a special interest in sleep disorders, and children Holly, 19, and Alisdair and Laurel, both 15.

Professor Hugh Malcolm Douglas Gurling, born 6 May 1950, died 2 November 2013.

